# Monitoring and Intervention Technologies to Manage Diabetic Older Persons: The CAPACITY Case—A Pilot Study

**DOI:** 10.3389/fendo.2020.00300

**Published:** 2020-05-13

**Authors:** Rodrigo Pérez-Rodríguez, Tania Guevara-Guevara, Pedro A. Moreno-Sánchez, Elena Villalba-Mora, Myriam Valdés-Aragonés, Myriam Oviedo-Briones, José A. Carnicero, Leocadio Rodríguez-Mañas

**Affiliations:** ^1^Biomedical Research Foundation, Getafe University Hospital, Getafe, Spain; ^2^Centre for Biomedical Technology, Universidad Politécnica de Madrid, Madrid, Spain; ^3^Geriatrics Service, Getafe University Hospital, Getafe, Spain

**Keywords:** diabetes, frailty, home-monitoring, prevention, early intervention

## Abstract

Diabetes Mellitus is a chronic disease with a high prevalence among older people, and it is related to an increased risk of functional and cognitive decline, in addition to classic micro and macrovascular disease and a moderate increase in the risk of death. Technology aimed to improve elder care and quality of life needs to focus in the early detection of decline, monitoring the functional evolution of the individuals and providing ways to foster physical activity, to recommend adequate nutritional habits and to control polypharmacy. But apart from all these core features, some other elements or modules covering disease-specific needs should be added to complement care. In the case of diabetes these functionalities could include control mechanisms for blood glucose and cardiovascular risk factors, specific nutritional recommendations, suited physical activity programs, diabetes-specific educational contents, and self-care recommendations. This research work focuses on those core aspects of the technology, leaving out disease-specific modules. These central technological components have been developed within the scope of two research and innovation projects (FACET and POSITIVE, funded by the EIT-Health), that revolve around the provision of integrated, continuous and coordinated care to frail older population, who are at a high risk of functional decline. Obtained results indicate that a geriatric multimodal intervention is effective for preventing functional decline and for reducing the use of healthcare resources if administered to diabetic pre-frail and frail older persons. And if such intervention is supported by the CAPACITY technological ecosystem, it becomes more efficient.

## Introduction

### Context

The World Health Organization (WHO) defines intrinsic capacity as the combination of the individual's physical and mental (including psychological) capacities. It is part of the functional ability together with the environment and the interactions with it. Frailty and intrinsic capacity are close and complementary concepts. Frailty might be perceived as a stage of the age-related decline of the physiological systems determining the reduction of the intrinsic capacity. A reduction that is also caused by other chronic diseases and conditions. When the functional reserve is highly narrowed, an increased risk of negative health outcomes occurs.

With the aging process, there are numerous physiological changes that increase the risk of developing chronic illnesses, disability, and dependency (1, 2). Therefore, healthcare systems need to shift to a person-centered care approach aimed at preventing declines on the intrinsic capacity. Early interventions are essential. Becoming frail or care dependent can be delayed, slowed or even partly or totally reversed. Furthermore, there is currently a pressing need to develop comprehensive community-based approaches, and to introduce interventions to prevent functional decline (3).

The overall estimated prevalence of frailty is 18% (95% CI: 15–21%). Longitudinal studies on aging have shown that frailty is correlated with age, gender (female) and socio-economic factors, in particular lower education and wealth (4). Frailty is reversible, and to do so it is necessary to act upon one of the main risk factors: inactivity and sedentariness (5). Activity-centered interventions have proven effective in delaying and even reversing frailty and disability (6–11). Other interventions include nutritional recommendations (12, 13) such as modifying eating habits, increasing protein and micronutrient intake, as well as interventions on polypharmacy and inadequate drug prescriptions (14–16), and interventions on psychological and social aspects (11). The Comprehensive Geriatric Assessment is the most efficient tool for the clinical management of those older patients at risk of frailty and disability; its holistic nature allows assessing the physical, functional, mental, and social sphere of the individuals.

As it happens with frailty, the incidence and prevalence of diabetes increases with age: more than 25% of the people aged ≥65 suffer from it. The International Diabetes Federation estimates that 18.6% of people aged 60–79 have diabetes. It is estimated that the number of diabetic persons aged 65–99 will be doubled by 2045. Similarly, the economic burden of diabetes will increase in the coming decades, especially among older groups (70–99), with an increase of 104 billion USD from 2017 to 2045 (17), mainly due to those with functional deterioration (18). Frailty can be associated with various chronic diseases, especially diabetes. Both conditions are highly prevalent in older individuals, share pathophysiological characteristics, and act synergistically to cause functional impairment in older adults. The prevalence of frailty is more pronounced in patients with diabetes mellitus, and the prevalence of diabetes mellitus is higher when frailty is present (19).

The joint association of physical inactivity and insulin resistance (i.e., a low level of insulin sensitivity) is associated with lower Skeletal Muscle Index, gait speed, and grip strength (20). A direct relationship between diabetes and risk of frailty has been found. Levels of glycosylated hemoglobin (HbA_1c_), a sedentary life-style, and low HDL-cholesterol are the main factors associated to this higher risk (21). Furthermore, consistent studies indicate that older people with diabetes are at higher risk of acute and chronic microvascular complications, that negatively impact their independency and self-caring capacity, leading to a worse quality of life (22).

In a study carried out with community dwelling population, authors conclude that frailty is an important risk factor for death and disability in diabetic older adults, supporting that frailty should be routinely assessed (23). A study that evaluated the progression of frailty and pre-frailty in an older cohort living in the community found that the likelihood of improving frailty was reduced in a 50% in diabetic pre-frail women (24). Cognitive impairment could dramatically affect the ability to self-manage diabetes, including poor glycemic control, and could represent a powerful prognostic factor to identify diabetic persons at high risk of mortality (25). This evidence suggests that diabetes is associated with poor prognostic factors in older persons.

Considering the complexity of diabetes and the burden of associated comorbidities in older people, frailty helps identifying diabetic older people as a highly vulnerable group that requires more careful and specific evaluations, as well as different therapeutic/intervention approaches aimed at alleviating or preventing functional deterioration. Strategies based on multicomponent physical activity demonstrated that a structured multimodal intervention program leads to a clinically relevant (and cost-effective) improvement in the functional status of pre-frail and frail elders with diabetes mellitus type 2 (5). Educational and nutritional interventions and the adjustment of HbA_1C_ targets also seem to be useful in preserving function and delaying disability in frail diabetic older patients (5, 26).

The frail older patient usually shows loss of neurological and muscle function. Besides, the definition of frailty revolves around the onset of accelerated weight loss with an associated decrease of the mass and strength in the skeletal muscle (27). Furthermore, in a systematic literature review it was found that, in a 30.6% of the selected studies, there was an association between gait speed and disability, frailty, sedentary lifestyle, falls, muscular weakness, diseases, body fat, cognitive impairment, mortality, stress, lower life satisfaction, lower quality of life, and poor performance in quantitative parameters of gait (28).

Given this context, it is of paramount importance to have fresh information related to certain variables associated to poor health outcomes. Monitoring the evolution of the gait speed, muscle power and involuntary weight loss may help defining preventive strategies to avoid disability. Information and Communication Technologies (ICTs) can play a crucial role in supporting these strategies, being specially effective the monitoring of vital signs and the provision of information through calls or educational contents (29). On the other hand, alert systems and fall detectors need further research to demonstrate evidence about their effectiveness (29).

Concerning frailty, home-based technology (30) as well as wearable sensors (31) or mHealth technology (32) may enable continuous and transparent monitoring of the independent elder, supporting the traditional geriatric approach to identify older people at risk of disability. However, Zaslavsky et al. (33) manifest that additional research needs to be conducted to evaluate not only the reliability and validity of the ICT-based measurement of frailty parameters (e.g., lower muscle functioning, unobtrusive energy expenditure, ADL performance, etc.), but also the ethical, technical, and economic issues behind it. Nevertheless, it should be considered that older population have already accepted new technologies, mainly because they have been proven useful in meeting their information needs, especially on health (34).

In general terms, technology needs to focus in (1) the early detection of functional decline, (2) monitoring the functional evolution of the elders providing ways to foster physical activity, (3) in providing recommendations of adequate nutritional habits, and (4) in controlling polypharmacy. But apart from all those functionalities, some other elements or modules covering disease-specific needs can be added to complement the care of the older individuals. In the specific case of diabetes, apart from detecting early functional impairment that could be related to further complications, added functionalities could include control mechanisms for blood glucose (with special attention to episodes of hypoglycemia) and cardiovascular risk factors, prognosis control, specific nutritional recommendations (e.g., adequate protein intake), suited physical activity (e.g., exercise programs including aerobic and endurance training), education on diabetes, self-care recommendations, and enhanced patient-healthcare communication tools (35).

There is an unprecedented need to improve the management of the healthcare provided to diabetic older persons, who are at an increased risk of falling due to diabetes complications, frailty or other conditions (36). New tools such as wearables (36, 37), environmental sensors (38), or gamification systems (39) may help identifying early risk indicators for adverse events and providing means for self-managing the disease. To this extent, mHealth technologies have demonstrated to be effective (35). In addition, involving older persons in the design and development process of such technologies is key to overcome usability barriers (40).

### Objective

This research work pursues evaluating the impact of delivering a multicomponent intervention supported by part of the CAPACITY system to a frail and diabetic older population.

This manuscript is structured as follows. First, the CAPACITY ecosystem is presented as a modular infrastructure to prevent disability. Second, the methodology is described to later present and discuss the obtained results. Finally, conclusions are extracted, and future work proposed.

## The Capacity Technological Ecosystem

CAPACITY is a technological ecosystem, developed jointly by Getafe University Hospital and Universidad Politécnica de Madrid, that lies on the technological substrate of two EU-funded (EIT-Health) research and innovation projects: FACET and POSITIVE. These 2 projects have produced a mature and low-cost home monitoring and remote intervention system aimed to prevent disability among the older population. FACET has allowed demonstrating that the home monitoring of variables with high predictive power for adverse events results in a fast improvement of the frailty status as well as in a reduction in the use of healthcare resources^1^. POSITIVE, currently ongoing in 2020, has completed the technological ecosystem by enabling a new organizational model that involves all relevant actors in the care process: older adults, informal caregivers and primary and specialized care professionals. POSITIVE will be deployed in 3 European countries with heterogeneous healthcare systems during 2020 and 2021.

CAPACITY's novel technological ecosystem allows shifting home and primary care settings conditions that are traditionally managed by specialized care. The proposed technological solution enables home monitoring of the intrinsic capacity, being the most relevant factors (those that can change in a short term) function, nutritional status, and state-of-mind of the older adults.

Using CAPACITY, older population can be remotely supervised by primary care professionals. If a dangerous decline in terms of the intrinsic capacity/frailty is detected, specialized care can be included into the loop (i.e., geriatricians). VIVIFRAIL experience, declared as success story by the European Commission (41), has been merged into the project's multifactorial treatment. VIVIFRAIL uses the Short Physical Performance Battery (SPPB), that assesses balance, gait speed, and time spent in standing-up and down from a chair, as a reference to automatically generate a tailored physical activity program to avoid disability. Multicomponent exercise programs for progressive muscle strengthening or generic strength training, balance retraining exercises, aerobic training, and flexibility training have been shown to improve critical outcomes.

**Figure 1:**
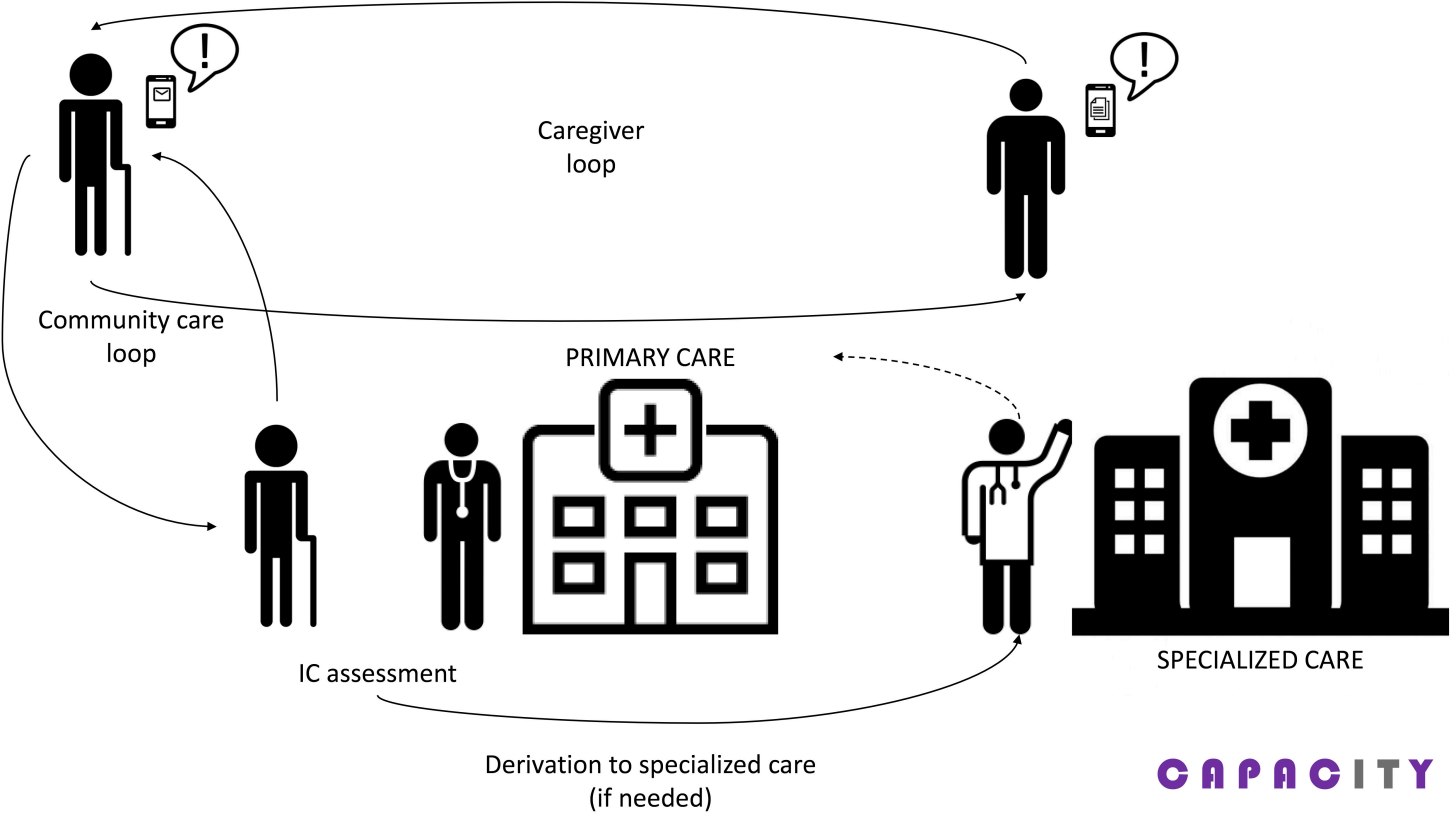
CAPACITY actors and interactions.

Figure 1 depicts all the actors and their interactions considered in the CAPACITY model. Older adults are involved in two care loops: one with the informal caregiver and another with primary care. The loop with specialized care is only created in case it is necessary (e.g., detection of decline).

CAPACITY, through a set of user-adapted mobile applications, offers the set of services described below. These services are aimed to keep older adults connected to the healthcare service provider and to his/her informal caregiver/s.

- Services offered to clinical professionals (primary and specialized care):
  ◦ Guidance to administer a Comprehensive Geriatric Assessment.
  ◦ Guidance to prescribe personalized preventive strategies.
  ◦ Management of alarms in case of dangerous decline that may lead to disability.
  ◦ Patients' follow-up based on the information collected by the home monitoring system.
  ◦ Tracking of the adherence to personalized interventions.
  ◦ Asynchronous communication with the cared seniors.
  ◦ Asynchronous communication with other clinical professionals.
- Services offered to older adults:
  ◦ Continuous monitoring of the intrinsic capacity, that may lead to triggering alarms indicating the potential need of attention protocols (aimed to prevent disability).
  ◦ Access to a personalized intervention (therapeutic plan).
  ◦ Access to the own evolution in terms of intrinsic capacity.
  ◦ Asynchronous communication with the healthcare service provider.
  ◦ Notifications on relevant alarms.
- Services offered to caregivers:
  ◦ Access to the evolution of the cared older person.
  ◦ Notifications on relevant alarms.

**Figure 2:**
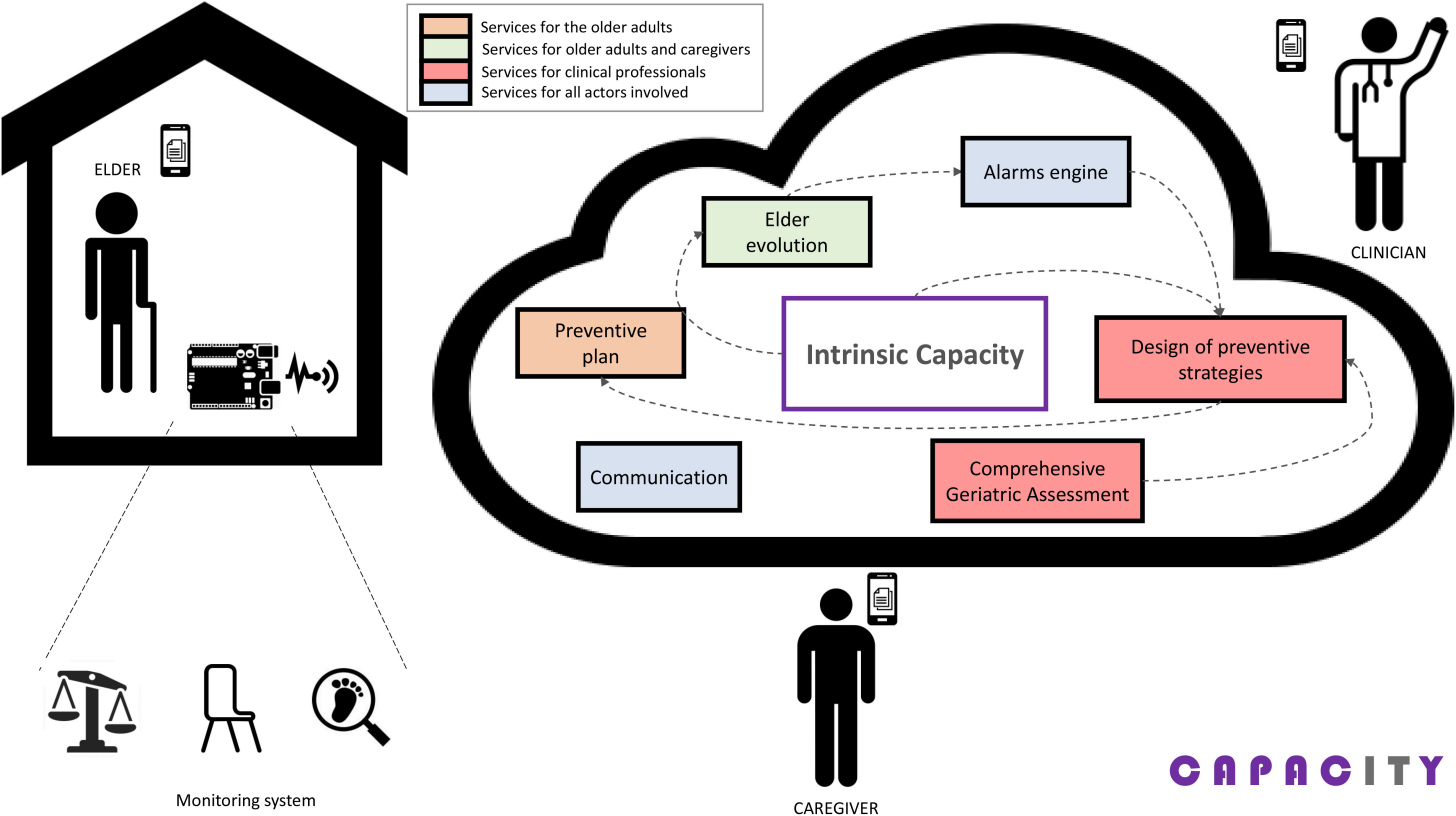
CAPACITY architecture.

Figure 2 shows all modules comprising the CAPACITY system. Table 1 maps the relationship of all these modules with the different services listed above, specifying the profile of the user and the means of access.

**Table 1:** Mapping between CAPACITY modules and services provided.

**Capacity module**	**Description**	**Service**	**User**	**How the service is delivered**
Comprehensive Geriatric Assessment	Clinical tests, questionnaires required to administer a full Comprehensive Geriatric Assessment to the older adults.	Guidance to administer a Comprehensive Geriatric Assessment	Clinician	App for clinical professionals
Design of preventive strategies	Prescription of personalized interventions: - Physical activity - Nutritional recommendations - Polypharmacy adaptations	Guidance to prescribe personalized preventive strategies	Clinician	
Preventive plan	Access to the personalized therapeutic plan: - VIVIFRAIL physical activity program through gamification - Nutritional recommendations - Pharmacological plan	Access to the personalized intervention	Older adult	App for older adults and CAPACITY monitoring kit
Monitoring system	Collection of variables to assess the evolution of the intrinsic capacity:	Continuous monitoring of the intrinsic capacity	Older adult	
	- Sensors to measure gait speed, power in the lower limbs, involuntary weight loss and level of physical activity - Treatment compliance. - Other variables collected through questionnaires (e.g., recent falls, state-of-mind, etc.)	Patients' follow-up based on the information collected by the Smart Home monitoring system, as well as adherence to the personalized intervention	Clinician	App for clinical professionals
Elder evolution	Graphic feedback displaying the evolution of the intrinsic capacity of the older adults	Access to the own evolution	Older adult	App for clinical professionals
		Access to the evolution of the cared elder	Caregiver	App for informal caregivers
Alarms engine	Processing of data provided by the home monitoring system to detect deterioration alarms and trigger early attention protocols aimed to avoid disability	Notifications on relevant alarmsManagement of alarms in case of dangerous decline that may lead to disability	Caregiver	App for clinical professionals
Communication	Communication services between actors to smooth the provision of healthcare attention	Asynchronous communication with the healthcare service provider	Older adult	App for older adults
		Asynchronous communication with the cared seniors	Clinician	App for clinical professionals
		Asynchronous communication with other clinical professionals		
Home automation	Proactive actions taken by the dwelling environment to avoid risky situations that may lead to disability	Home automation to avoid potential disability	Older adult	App for older adults

**Figure 3:**
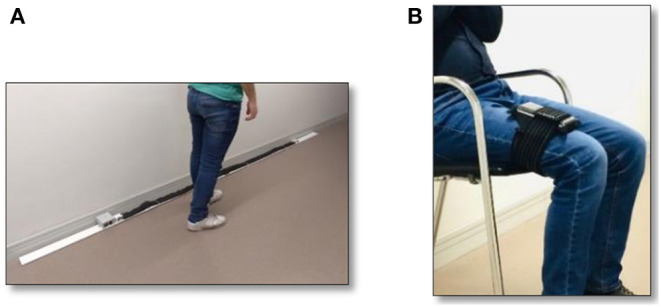
Current monitoring kit prototypes: **(A)** gait speed sensor and **(B)** sensor to measure power.

The purpose of the home monitoring system is to collect relevant information that is generated out of the clinical environment. Further processing may lead triggering early attention protocols if necessary. This monitoring system consists in a set of Internet of Things devices (monitoring kit) that measure variables with high prediction power for adverse events. The home monitoring kit consists in a gait speed sensor, a sensor to measure power in the lower limbs, and a wireless commercial weight scale to measure involuntary weight loss. Figure 3 illustrates the prototypes of the sensors specifically designed for CAPACITY, that are currently (in 2020) under an industrialization process to improve their encapsulation and look and feel.

The home monitoring kit is complemented by a set of simplified and adapted questionnaires (accessed through a mobile application) to assess self-perception of health, loss of appetite, fatigability, physical resistance, ambulation, state of mind, pain, and fall events.

## Methodology

The impact of administering a multicomponent intervention partially supported by the CAPACITY ecosystem has been assessed by carrying out a sub-study only considering diabetic prefrail and frail individuals participating in the experimental stage of the FACET project. FACET conducted a pilot, prospective, randomized, and blind study. The pilot study lasted 12 months: 6 months were dedicated to recruitment, and 6 months to intervention. Diabetes was assessed by clinical diagnosis (clinical history, antidiabetic drugs) and self-reported comorbidity through the Charlson Index (42).

FACET primary and secondary objectives were:

- Primary:
  ◦ To evaluate whether the FACET system avoid transitions from pre-frailty to frailty according to the Fried's Criteria (43), Frail Scale (44), and Frailty Trait Scale 5 (FTS-5), that is a reduced version for of the FTS (45).
  ◦ To evaluate whether the FACET system improves frailty in at least 1 point in the Fried's Criteria, 1 point in the Frail Scale and 2,5 points in the FTS-5.
- Secondary:
  ◦ To evaluate whether the FACET system improves patients' physical performance as measured by SPPB, gait speed, and Chair Stand Test.
  ◦ To evaluate the influence of the FACET system in the cognitive sphere as measured as MMSE, semantic and phonologic fluency, and Clock Drawing Test.
  ◦ To evaluate the influence of the FACET system in the affective sphere as measured as the Geriatric Depression Scale.
  ◦ To evaluate the influence of the FACET system on the quality of life as measured as the EURO-QoL 5D-5L.
  ◦ To evaluate the influence of the FACET system on the number of falls suffered during the intervention.
  ◦ To evaluate the influence of the FACET system on the use of health resources.

To determine the sample size, since no previous similar studies were found, a recruitment objective of 90 pre-frail and frail older individuals was set and fully accomplished. This figure comes from the following reasoning:

- Since 2 group of interest were identified (frail and pre-frail), 20 individuals per group and research arm was considered adequate for a pilot study according to the commonly used standards.
- A 10–15% loss was initially estimated.

Two research groups (arms) were defined within the validation study. A control group (*n* = 44) receiving usual geriatric care and an intervention group (*n* = 46) who received the same multicomponent intervention but partially supported by the CAPACITY system. Frail participants were over-sampled (53%) to compensate their potentially higher dropout rate. Pre-frail participants were those meeting 2 Linda Fried's criteria and suffering from at least 4 comorbidities, since they are the ones with the highest risk for developing frailty. Frail individuals were those meeting at least 3 Linda Fried's criteria and having at least 4 comorbidities. Stratification by age (70–85, >85), gender (male, female), diagnosis (frail and pre-frail), and education (higher education, not formal education, others) was applied to guarantee the groups were comparable. Participants were assessed at baseline, after 3 months of follow-up and at the end of the intervention (6 months after recruitment). Recruitment, assessment, follow-up, and treatment was carried out parallelly in 2 institutions: Getafe University Hospital and Albacete University Hospital.

The CAPACITY modules that supported the intervention delivered to the participants in the intervention group were: (1) monitoring system, (2) elder evolution, and (3) basic asynchronous communication.

If the clinician detected any relevant alterations in the evolution of any participant randomized into the intervention group, the individual was directly contacted. The older person could also contact the doctor at any time through the basic asynchronous communication module.

Descriptive statistics are presented within this manuscript as mean (std) and N (proportions). Linear and logistic regression methods were used to assess the primary and secondary objectives.

## Results

Ninety participants were recruited (44 randomized into the control group and 46 into the intervention group). Mean age was 82 years, 72% were women. From the whole sample, 26 participants were diabetic (14 in the control group and 12 in the intervention group), mean age of 84 years, 65% were women, 39% did not receive formal education, and 58% did not have any experience with technology. The research presented in this manuscript only focuses on the diabetic population.

The effect of diabetes in improving 2,5 points in frailty measured with FTS-5 has been studied separately for each research arm (models based on Linda Fried's Criteria could not run due the reduced number of diabetic older persons improving in such test). In both groups, diabetes is a restraining factor for improving frailty status. Obtained results are OR = 0.25 (95% CI = 0.04–1.46; p-v = 0.12) for the control group and OR = 0.14 (95% CI = 0.01–1.28; p-v = 0.081) for the intervention group.

A statistically significant association in the improvement of the frailty status measured with FTS-5 between baseline and the third month of follow-up, OR = 7.9 (95% CI = 1.1–56.1; p-v = 0.039), has been found for the intervention group in relation to the control group. In terms of Linda Fried's Criteria, a trend on the improvement was found, OR = 3 (95% CI = 0.31–28.84; p-v = 0.34).

It has been also found statistically significant differences in the improvement of frailty using Linda Fried's Criteria between baseline and the sixth month of follow-up for both research arms (control group, p-v: 0.037; intervention group, p-v: 0.044). In terms of FTS-5 after 6 months of follow-up, results are not statistically significant, but an improvement trend is observed (control group, p-v: 0.16 -average change 1.86 points-; intervention group, p-v: 0.13 -average change 2 points-).

Table 2 compares the use of healthcare resources depending on the research arm diabetic subjects were randomized into. Participants allocated into the intervention group visited the primary care nurse 0.7 fewer times those randomized into the control group did (beta: −0.72; 95% CI = −1.1–(−0.3); p-v = 0.0002), visited the primary care doctor 0.9 fewer times (beta: −0.91; 95% CI = −1.3–(−0.518); p-v = 5.298E-06) and spent 0.9 fewer days in the hospital after admission than the subjects pertaining to the control group (beta: 0.9; 95% CI = 0.159–(1.65); p-v = 0.017).

**Table 2:** Difference in the use of healthcare resources between the intervention and control groups.

**Healthcare resource**	***p***	**OR**	**95% CI**
Falls	0.41	0.424	0.025-4.319
Number of hospital admissions	0.493	0.409	0.031-5.276
**Healthcare resource**	***p***	**beta**	**95% CI**
Number of visits to the emergency room	0.375	0.392	−0.476-1.261
**Number of days of hospitalization**	**0.017**	**0.907**	**0.159-1.655**
**Number of visits to the primary care doctor**	**5.298E-06**	**−0.910**	**−1.302-(−0.518)**
**Number of visits to the primary care nurse**	**0.0002**	**−0.718**	**−1.093-(−0.344)**
Number of visits to specialist	0.08	−0.417	−0.860-0.049

*Bolded values are statistically significant results*.

## Discussion

Recruitment data agrees with current prevalence data. Out of the pre-frail and frail sample, 29% were diabetic, outlining the importance of targeting such population (19). Besides, obtained results indicate that the geriatric multimodal intervention administered with and without the support of the CAPACITY system is effective to improve the frailty status among the diabetic older population, what is directly related to a reduction in the risk for adverse events.

It is important to remark that although frailty improves earlier (at month 3) in those subjects allocated in the intervention group, controls also show improvement later in the follow-up (at month 6). The reason behind this can be associated to the fact that all participants, regardless the research arm they were randomized into, underwent a multimodal intervention designed by a geriatrician. Improvements were expected regardless the research group. The study did not try to assess the effectiveness of the intervention against placebo: control group received usual care for frail patients. This fact accounts for the improvement in the control branch. Accordingly, the intervention provided an additional benefit to that provides by usual care.

Findings confirm what Rodríguez-Mañas et al. already demonstrated (5). But if the multmodal intervention is supported by the CAPACITY technological ecosystem, multimodal interventions become more efficient (individuals improve faster). Moreover, it has been also found that suffering from diabetes makes improving frailty status harder, which is in line with the findings by Castro-Rodríguez et al. and Lee et al., that confirmed diabetes as an important risk factor for adverse events (23, 24).

Regarding the use of healthcare resources by the diabetic subpopulation, results demonstrate that the number of visits to the primary care doctor and nurse as well as the length of hospital stay get reduced for those receiving the multimodal intervention supported by the CAPACITY system. On the other hand, although not statistically significant, promising trends have been found in terms of number of falls, number of hospital admissions, number of visits to the emergency department and number of visits to the specialist. These not statistically significant data may be due to the small sample size. However, results seem to indicate that the CAPACITY-supported multimodal intervention, apart from being more efficient from the clinical perspective, it is also in terms of use of healthcare resources, which might mitigate current prospects on the healthcare expenditure of those older diabetic individuals (17).

## Conclusions

This research work has confirmed that a geriatric multimodal intervention based on a multicomponent exercise program, nutritional recommendations, and polypharmacy regulation is effective for preventing functional decline (significantly reducing the risk for adverse events) and for reducing the use of healthcare resources if administered to diabetic pre-frail and frail older individuals. On top of that, this multimodal intervention gets even more effective when it is partially supported by the CAPACITY ecosystem. Better results are expected when the intervention is fully supported by the proposed solution (i.e., deploying and using all modules described in section The CAPACITY Technological Ecosystem).

Furthermore, the fact of diabetes being a restraining factor for improving frailty, supports the authors' hypotheses on the need of complementing the CAPACITY technological ecosystem with new disease-specific modules aimed to equate the effect of the geriatric multimodal intervention, alleviating this restraint (e.g., control mechanisms for blood glucose and cardiovascular risk factors, specific nutritional recommendations, a suited physical activity program, diabetes-specific educational contents, self-care recommendations, etc.).

However, a lack of statistical power due to the small sample size is reflected in wide confidence intervals. This uncertainty may limit the authors' conclusions. Besides, findings based on a follow-up period of 6 months may not be considered definitive.

Future work will include carrying out further experimentation to mitigate the limitations of this study and confirm obtained promising results. A wider sample of subjects with diabetes will be recruited (the amount of diabetic pre-frail and frail individuals among the overall population was scarce). Also, follow-up time will be extended. Besides, the CAPACITY system will be evolved to cover the needs of the diabetic pre-frail and frail older individuals.

## Data Availability Statement

The original contributions presented in the study are included in the article/supplementary material, further inquiries can be directed to the corresponding author/s.

## Ethics Statement

The studies involving human participants were reviewed and approved by Getafe University Hospital Ethical Committee. The patients/participants provided their written informed consent to participate in this study.

## Author Contributions

LR-M generated the original idea and coordinated the team. RP-R, PM-S, and EV-M conceptualized and materialized the technological approach. MV-A, RP-R, PM-S, MO-B, and LR-M designed the trial. RP-R led and supervised the correct execution of the experiment, with support from PM-S, MV-A, MO-B, and EV-M. JC performed the statistical analysis. TG-G, MV-A, and LR-M analyzed the clinical results. All authors participated in writing the manuscript.

## Conflict of Interest

The authors declare that the research was conducted in the absence of any commercial or financial relationships that could be construed as a potential conflict of interest. The handling Editor declared a past co-authorship with one of the authors LR-M.

## Footnotes

^1^Results in process of publication at the moment of writing this manuscript.

## References

[1] Fulop T Larbi A Witkowski JM McElhaney J Loeb M Mitnitski A. Aging, frailty and age-related diseases. Biogerontology. (2010) 11:547–63. 10.1007/s10522-010-9287-2 20559726

[2] Rodriguez-Mañas L Fried LP. Frailty in the clinical scenario. Lancet. (2015) 385:e7–9. 10.1016/S0140-6736(14)61595-6 25468154

[3] WHO Integrated Care for Older People: Guidelines on Community-Level Interventions to Manage Declines in Intrinsic Capacity (2017). 29608259

[4] O'Caoimh R Galluzzo L Rodríguez-Laso Á der Heyden J Ranhoff AH Lamprini-Koula M. Prevalence of frailty at population level in European ADVANTAGE Joint Action Member States: a systematic review and meta-analysis. Ann Ist Super Sanita. (2018) 54:226–39. 10.4415/ANN_18_03_10 30284550

[5] Rodriguez-Mañas L Laosa O Vellas B Paolisso G Topinkova E Oliva-Moreno J. Effectiveness of a multimodal intervention in functionally impaired older people with type 2 diabetes mellitus. J Cachexia Sarcopenia Muscle. (2019) 10:721–33. 10.1002/jcsm.12432 31016897 PMC6711410

[6] Izquierdo M Casas-Herrero A Martínez-Velilla N Alonso-Bouzón C Rodríguez-Mañas L. An example of cooperation for implementing programs associated with the promotion of exercise in the frail elderly. European Erasmus+guillemotleft}Vivifrail{/guillemotright} program. Rev Esp Geriatr Gerontol. (2017) 52:110–1. 10.1016/j.regg.2016.03.004 27132063

[7] IzquierdoMRodriguez-MañasLSinclairAJVivifrail Investigators Group. What is new in exercise regimes for frail older people — How does the Erasmus Vivifrail Project take us forward? J Nutr Heal Aging. (2016) 20:736–7. 10.1007/s12603-016-0702-527499307

[8] Seldeen KL Lasky G Leiker MM Pang M Personius KE Troen BR. High Intensity Interval Training (HIIT) improves physical performance and frailty in aged mice. J Gerontol A Biol Sci Med Sci. (2018) 73:429–37. 10.1016/j.mad.2019.04.001 28633487

[9] Silva RB Aldoradin-Cabeza H Eslick GD Phu S Duque G. The effect of physical exercise on frail older persons: a systematic review. J Frailty Aging. (2017) 6:91–6. 10.14283/jfa.2017.7 28555710

[10] Makizako H Shimada H Doi T Tsutumimoto K Yoshida D Suzuki T. Effects of a community disability prevention program for frail older adults at 48-month follow up. Geriatr Gerontol Int. (2017) 17:2347–53. 10.1111/ggi.13072 28627050

[11] Dedeyne L Deschodt M Verschueren S Tournoy J Gielen E. Effects of multi-domain interventions in (pre)frail elderly on frailty, functional, and cognitive status: a systematic review. Clin Interv Aging. (2017) 12:873–96. 10.2147/CIA.S130794 28579766 PMC5448695

[12] Yannakoulia M Ntanasi E Anastasiou CA Scarmeas N. Frailty and nutrition: from epidemiological and clinical evidence to potential mechanisms. Metabolism. (2017) 68:64–76. 10.1016/j.metabol.2016.12.005 28183454

[13] Cruz-Jentoft AJ Kiesswetter E Drey M Sieber CC. Nutrition, frailty, and sarcopenia. Aging Clin Exp Res. (2017) 29:43–8. 10.1007/s40520-016-0709-0 28155181

[14] Herr M Sirven N Grondin H Pichetti S Sermet C. Frailty, polypharmacy, and potentially inappropriate medications in old people: findings in a representative sample of the French population. Eur J Clin Pharmacol. (2017) 73:1165–72. 10.1007/s00228-017-2276-5 28601963

[15] Maclagan LC Maxwell CJ Gandhi S Guan J Bell CM Hogan DB. Frailty and potentially inappropriate medication use at nursing home transition. J Am Geriatr Soc. (2017) 65:2205–12. 10.1111/jgs.15016 28752589

[16] Veronese N Stubbs B Noale M Solmi M Pilotto A Vaona A. Polypharmacy is associated with higher frailty risk in older people: an 8-year longitudinal cohort study. J Am Med Dir Assoc. (2017) 18:624–8. 10.1016/j.jamda.2017.02.009 28396180 PMC5484754

[17] International Diabetes Federation IDF Diabetes Atlas Eighth edition 2017. IDF Diabetes Atlas, 8th ed Brussels: International Diabetes Federation (2017). Available online at: http://www.diabetesatlas.org

[18] Rodríguez-Sánchez B Angelini V Feenstra T Alessie RJM. Diabetes-associated factors as predictors of nursing home admission and costs in the elderly across Europe. J Am Med Dir Assoc. (2017) 18:74–82. 10.1016/j.jamda.2016.09.011 27815109

[19] Sinclair AJ Abdelhafiz AH Rodríguez-Mañas L. Frailty and sarcopenia - newly emerging and high impact complications of diabetes. J Diabetes Compl. (2017). 31:1465–73. 10.1016/j.jdiacomp.2017.05.003 28669464

[20] Yang CW Li CI Li TC Liu CS Lin CH Lin WY. The joint association of insulin sensitivity and physical activity on the skeletal muscle mass and performance in community-dwelling older adults. Exp Gerontol. (2017) 95:34–8. 10.1016/j.exger.2017.05.006 28502778

[21] García-Esquinas E Graciani A Guallar-Castillón P López-García E Rodríguez-Mañas L Rodríguez-Artalejo F. Diabetes and risk of frailty and its potential mechanisms: a prospective cohort study of older adults. J Am Med Dir Assoc. (2015) 16:748–54. 10.1016/j.jamda.2015.04.008 25986874

[22] Sue Kirkman M Briscoe VJ Clark N Florez H Haas LB Halter JB. Diabetes in older adults: a consensus report. J Am Geriatr Soc. (2012) 60:2342–56. 10.1111/jgs.12035 23106132 PMC4525769

[23] Castro-Rodríguez M Carnicero JA Garcia-Garcia FJ Walter S Morley JE Rodríguez-Artalejo F. Frailty as a major factor in the increased risk of death and disability in older people with diabetes. J Am Med Dir Assoc. (2016) 17:949–55. 10.1016/j.jamda.2016.07.013 27600194

[24] Lee JSW Auyeung TW Leung J Kwok T Woo J. Transitions in frailty states among community-living older adults and their associated factors. J Am Med Dir Assoc. (2014) 15:281–6. 10.1016/j.jamda.2013.12.002 24534517

[25] Thein FS Li Y Nyunt MSZ Gao Q Wee SL Ng TP. Physical frailty and cognitive impairment is associated with diabetes and adversely impact functional status and mortality. Postgrad Med. (2018) 130(6):561–7. 10.1080/00325481.2018.1491779 29949390

[26] Assar M El Laosa O Rodríguez Mañas L. Diabetes and frailty. Curr Opin Clin Nutr Metab Care. (2019) 22:52–7. 10.1097/MCO.0000000000000535 30394893

[27] Kinney JM. Nutritional frailty, sarcopenia and falls in the elderly. Curr Opin Clin Nutr Metab Care. (2004). 7:15–20. 15090898 10.1097/00075197-200401000-00004

[28] Binotto MA Lenardt MH Rodríguez-Martínez MDC. Physical frailty and gait speed in community elderly: a systematic review. Rev Esc Enferm USP. (2018) 52:e03392. 10.1590/S1980-220X2017028703392 30570081

[29] Barlow J Singh D Bayer S Curry R. A systematic review of the benefits of home telecare for frail elderly people and those with long-term conditions. J Telemed Telecare. (2007). 13:172–9. 10.1258/135763307780908058 17565772

[30] Lunardini F Luperto M Romeo M Renoux J Basilico N Krpic A The MOVECARE Project: Home-based Monitoring of Frailty. In: IEEE EMBS International Conference on Biomedical & Health Informatics (BHI). Chicago, IL: IEEE (2019). p. 1–4.

[31] Mohler MJ Wendel CS Taylor-Piliae RE Toosizadeh N Najafi B. Motor performance and physical activity as predictors of prospective falls in community-dwelling older adults by frailty level: application of wearable technology. Gerontology. (2016) 62:654–64. 10.1159/000445889 27160666 PMC5073011

[32] Changizi M Kaveh MH. Effectiveness of the mHealth technology in improvement of healthy behaviors in an elderly population—A systematic review. mHealth. (2017) 3:51. 10.21037/mhealth.2017.08.06 29430455 PMC5803024

[33] Zaslavsky O Thompson H Demiris G. The role of emerging information technologies in frailty assessment. Res Gerontol Nurs. (2012). 5:216–28. 10.3928/19404921-20120410-02 22533942

[34] Zhou J Rau PLP Salvendy G Age-related difference in the use of mobile phones. Univ Access Inform Soc. (2014) 13:401–3. 10.1007/s10209-013-0324-1

[35] Valencia WM Botros D Vera-Nunez M Dang S. Diabetes treatment in the elderly: incorporating geriatrics, technology, and functional medicine. Curr Diabetes Rep. (2018) 18:95. 10.1007/s11892-018-1052-y 30187176

[36] Najafi B Armstrong DG Mohler J. Novel wearable technology for assessing spontaneous daily physical activity and risk of falling in older adults with diabetes. J Diabetes Sci Technol. (2013). 7:1147–60. 10.1177/193229681300700507 24124940 PMC3876357

[37] Miller JD Najafi B Armstrong DG Current standards and advances in diabetic ulcer prevention and elderly fall prevention using wearable technology. Curr Geriatr Rep. (2015) 4:249–56. 10.1007/s13670-015-0136-7

[38] Chatterjee S Byun J Pottathil A Moore MN Dutta K Xie H Persuasive sensing: a novel in-home monitoring technology to assist elderly adult diabetic patients. In: International Conference on Persuasive Technology. Springer (2012). p. 31–42.

[39] Lee S Shin S. Effectiveness of virtual reality using video gaming technology in elderly adults with diabetes mellitus. Diabetes Technol Ther. (2013) 15:489–96. 10.1089/dia.2013.0050 23560480

[40] Isaković M Sedlar U Volk M Bešter J. Usability pitfalls of diabetes mHealth apps for the elderly. J Diabetes Res. (2016). 2016:1604609. 10.1155/2016/1604609 27034957 PMC4807066

[41] Promote Physical Exercise in Frail Elderly. (2016) Available online at: https://ec.europa.eu/programmes/erasmus-plus/projects/eplus-project-details/#project/9d8fc2db-47ad-429c-8b37-e2c210138339

[42] Charlson ME Pompei P Ales KL MacKenzie CR. A new method of classifying prognostic comorbidity in longitudinal studies: development and validation. J Chronic Dis. (1987) 40:373–83. 3558716 10.1016/0021-9681(87)90171-8

[43] Fried LP Tangen CM Walston J Newman AB Hirsch C Gottdiener J Frailty in older adults : evidence for a phenotype. J Geronterol. (2001) 56A:M146–57. 10.1093/gerona/56.3.M146 11253156

[44] Morley JE Malmstrom TK Miller DK. A simple frailty questionnaire (FRAIL) predicts outcomes in middle aged african americans. J Nutr Heal Aging. (2012) 16:601–8. 10.1007/s12603-012-0084-2 22836700 PMC4515112

[45] García-García FJ Carcaillon L Fernandez-Tresguerres J Alfaro A Larrion JL Castillo C. A new operational definition of frailty: the frailty trait scale. J Am Med Dir Assoc. (2014) 15:371–7. 10.1016/j.jamda.2014.01.004 24598478

